# Eye Movements during Auditory Attention Predict Individual Differences in Dorsal Attention Network Activity

**DOI:** 10.3389/fnhum.2016.00164

**Published:** 2016-05-09

**Authors:** Rodrigo M. Braga, Richard Z. Fu, Barry M. Seemungal, Richard J. S. Wise, Robert Leech

**Affiliations:** ^1^Computational, Cognitive and Clinical Neuroimaging Laboratory, Division of Brain Sciences, Imperial College London, Hammersmith Hospital CampusLondon, UK; ^2^Center for Brain Science, Harvard UniversityCambridge, MA, USA; ^3^Aathinoula A. Martinos Center for Biomedical ImagingCharlestown, MA, USA

**Keywords:** auditory attention, listening, eye movements, saccades, dorsal attention network, spatial attention

## Abstract

The neural mechanisms supporting auditory attention are not fully understood. A dorsal frontoparietal network of brain regions is thought to mediate the spatial orienting of attention across all sensory modalities. Key parts of this network, the frontal eye fields (FEF) and the superior parietal lobes (SPL), contain retinotopic maps and elicit saccades when stimulated. This suggests that their recruitment during auditory attention might reflect crossmodal oculomotor processes; however this has not been confirmed experimentally. Here we investigate whether task-evoked eye movements during an auditory task can predict the magnitude of activity within the dorsal frontoparietal network. A spatial and non-spatial listening task was used with on-line eye-tracking and functional magnetic resonance imaging (fMRI). No visual stimuli or cues were used. The auditory task elicited systematic eye movements, with saccade rate and gaze position predicting attentional engagement and the cued sound location, respectively. Activity associated with these separate aspects of evoked eye-movements dissociated between the SPL and FEF. However these observed eye movements could not account for all the activation in the frontoparietal network. Our results suggest that the recruitment of the SPL and FEF during attentive listening reflects, at least partly, overt crossmodal oculomotor processes during non-visual attention. Further work is needed to establish whether the network’s remaining contribution to auditory attention is through covert crossmodal processes, or is directly involved in the manipulation of auditory information.

## Introduction

The visual and auditory sensory systems can be thought of as serving a single role—to gather information about our surroundings so that we may adapt our behavior accordingly. When a loud sound alerts us to a potentially dangerous situation, our eyes instinctively orient towards the source of that sound to gain further knowledge of its identity. This example highlights that the two sensory systems are intimately linked, with attention-capture in one modality often leading to recruitment of the other. Despite this, we are able to control which features of a given sensory modality we wish to pay attention to. This suggests some degree of modal separation in the “top-down” or “endogenous” attentional modulation of sensory information. This nuanced relationship poses a conundrum for establishing the neural correlates of auditory and visual attention, if indeed they are subserved by separate systems.

In vision, a great body of research has established that a dorsal frontoparietal network comprised of the superior parietal lobes (SPL) and frontal eye fields (FEF) is activated during top-down attention (Kincade et al., 2005; Vossel et al., 2006; Corbetta et al., 2008). This network is sometimes called the “dorsal attention network” (DAN; Corbetta et al., 2008), and is active for example during the maintenance of attention to a visual field location in anticipation of a visual stimulus (Kastner et al., 1999). There is complementary evidence that the core nodes of this network, the SPL and FEF, do have a primarily visuospatial role. The regions are known to be involved in eye movement control (Büttner-Ennever and Horn, 1997) and visuospatial processing (Behrmann et al., 2004). Retinotopic maps have been located in both the FEF and SPL using direct stimulation and functional neuroimaging (Moore et al., 2003; Ruff et al., 2008; Saygin and Sereno, 2008). The FEF and neighboring supplementary eye fields (SEF) were shown to be activated during the planning of subsequent saccades using electrophysiology and functional magnetic resonance imaging (fMRI; Isoda and Tanji, 2003; Hu and Walker, 2011). Further, transcranial magnetic stimulation (TMS) of the FEF delays voluntary saccades (Muggleton et al., 2011). These different lines of evidence converge on the DAN having a role in oculomotor control and visual orienting.

In hearing, the networks subserving top-down attention are not as well understood. A frontotemporal network consisting of the middle and inferior frontal gyri (IFG) and regions near the posterior superior temporal sulcus (pSTS), has been proposed to mediate the orienting of attention to non-spatial features of sounds, such as frequency and identity (Maeder et al., 2001; Salmi et al., 2007; Braga et al., 2013; Seydell-Greenwald et al., 2013). In support of this, activity in the SPL and FEF is notably absent from tasks that require auditory attention such as speech and music perception (Hickok et al., 2003; Warren, 2008). However, during sound localization dorsal frontoparietal activity is often observed (e.g., Alho et al., 2015) even in the absence of visual stimuli (Lewis et al., 2000; Alain et al., 2001; Maeder et al., 2001; Shomstein and Yantis, 2006; Petit et al., 2007; Hill and Miller, 2010). The FEF even shows preparatory activity for spatial listening in the absence of auditory or visual stimuli (Lee et al., 2013). This has led to the theory that the dorsal frontoparietal network is “amodal” and directly mediates attentional orienting to all sensory modalities (Posner and Petersen, 1990; Driver and Spence, 1998; Macaluso, 2010).

It is difficult to reconcile the DAN’s role in eye movement control with its recruitment during auditory orienting without recourse to a possible crossmodal cause (Driver and Spence, 1998). Auditory attention is likely to involve both direct modality-specific as well as indirect cross-modal processes, and the role of the DAN in this regard is not clear. On the one hand, activation of the DAN could represent direct manipulation of auditory processes, such as the top-down tuning of auditory spatial receptive fields in the auditory cortex (Fritz et al., 2010). Alternatively, the activation of the DAN during listening may be representative of indirect processes such as visual spatial orienting or task-induced eye movements. Such processes may be facilitatory for auditory attention, even if not directly involved in modulating auditory information.

There is behavioral evidence that auditory attention elicits systematic eye movements (Paulsen and Ewertsen, 1966; Rolfs et al., 2005; Valsecchi and Turatto, 2009; Kerzel et al., 2010; Yuval-Greenberg and Deouell, 2011; Zou et al., 2012). For example, an auditory stimulus leads reliably to visual saccades towards the source of the sound (Zahn et al., 1978; Zambarbieri et al., 1982; Van Grootel and Van Opstal, 2009). Rotating a sound about a subject’s head can induce nystagmus (Paulsen and Ewertsen, 1966). Further, the presentation of an auditory stimulus can reduce the rate of saccades (Rolfs et al., 2005; Kerzel et al., 2010; Yuval-Greenberg and Deouell, 2011; Zou et al., 2012). There is also evidence that gaze position can affect auditory localization accuracy (Maddox et al., 2014). However, neuroimaging studies rarely consider the influence of eye movements on auditory attention, meaning that parts of the networks implicated in auditory attention may in fact be mediating these crossmodal effects.

**Figure 1:**
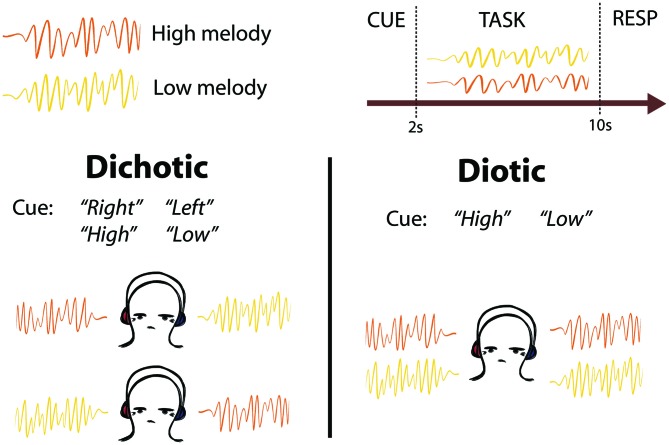
**Schematic of task design.** Two competing melodies were presented simultaneously using either dichotic (one melody played to each ear) or diotic (both melodies played to both ears equally) listening. Subjects were cued to listen to the melody on the left or right ear (“Left” or “Right”), or to listen to the melody that was higher or lower in pitch (“High” or “Low”). After the melodies were completed, subjects were cued to Respond (RESP) with button presses to indicate whether they heard a pitch change in the cued melody.

Given that the DAN, and particularly the FEF, is known to be involved in the generation of saccades, it is possible that its recruitment during listening tasks reflects task-induced indirect oculomotor processes. To test this hypothesis, eye movements need to be recorded in an auditory attention task in the absence of visual stimuli and without any requirement for saccades or fixation. In contrast, the usual approach has been to employ fixation conditions to investigate how gaze position affects auditory processing (e.g., sound localization performance; Maddox et al., 2014). If DAN activity during listening is associable with indirect processes such as increased eye movement control while other parts of the auditory network are not, this would provide evidence that DAN activation is the result of an indirect attentional route. This evidence would be particularly strong if the magnitude of DAN activity corresponds with the magnitude of indirect crossmodal influences. In the present manuscript we recorded eye movements during a purely auditory attention task using fMRI and in-scanner eye-tracking (Figure 1). Our hypothesis was that attentive listening would be associated with systematic effects on eye movements, and that these effects would be associated with the magnitude of activity within visuospatial regions of the DAN.

## Materials and Methods

### Subjects

Twenty healthy right-handed volunteers (9 female, mean age 26.2, range 21–36). All participants reported no hearing problems and had normal or corrected vision (via contact lenses or MRI compatible glasses). The study was conducted in accordance with the guidelines of Imperial College Research Ethics Committee, and written consent was obtained from all volunteers before their participation. Participants were screened for contraindications to MRI, and were excluded on the basis of hearing difficulties and previous psychiatric or neurological disorders. Four additional participants were scanned but had to be excluded due to technical issues with the eye tracking equipment (3) and excessive motion (1).

### Auditory Task

Subjects listened to 12 different looped melodies. Each melody lasted 2 s, and was repeated four times in each trial so that each trial lasted 8 s. The melodies were generated by manually selecting sequences of diatonic notes within one octave using prepackaged synthesizer sounds from the Logic Pro X Software (version 10.2.2). Each melody contained between 7 and 12 staccato notes (mean 10 notes). Six of the melodies had a low tonal center (F above C1), and six had a high tonal center (C3), with no overlap in pitch between high and low pitch sequences. In each trial, two looped melodies, one high and one low, were presented simultaneously, either dichotically (one sound in each ear) or diotically (both sounds in both ears equally; Figure 1). The competing melodies overlapped in terms of note onset and duration, but not pitch. Stimuli were presented using Sensimetrics S14 sound-attenuating in-ear MR-compatible headphones. Subjects were trained outside the scanner to listen out for an oddball target in the form of a “pitch change”, which was in fact a transposition of the whole 2 s melody to a tonal center 7 semitones above the original key. This key change made the two melodies incongruous and the target detectable. The task was split into two identical blocks. Each block contained 70 trials, of which 23 contained a pitch change in the cued melody (the target), seven contained a distractor pitch change in the non-cued melody (catch trials), and 10 were silent rest trials. Targets were not presented in 30/70 trials. Targets and distractors were presented in either the second (7/70), third (9/70) or fourth (14/70) repeat of the 2 s diatonic melody, in a pseudo-randomized order to avoid long repeats and ensure an even distribution of Rest trials. Distractors and targets were never presented in the same trial. The order of stimulus presentation was changed halfway through the experiment (6 subjects received one order and 14 received the other) to control for order-effects. We performed a confirmatory analysis with balanced groups (*n* = 6 subjects receiving each presentation order) which confirmed that the eye movement behavioral results reported were not due to order-effects. Before each trial, subjects were presented with a diotic auditory spoken word (“Right”, “Left”, “High” or “Low”) which cued them to listen to the melody presented in their right or left ear, or that was higher or lower in pitch, respectively. The cue period lasted 2 s, and the spoken cue onset was at the start of those 2 s (not centered within the 2 s). The task period lasted 8 s, and the response period lasted 3 s (including an auditory “Please respond” cue). Each trial was followed by a period of silence lasting between 1–3 s. Ten silent “Rest” trials were also interspersed between listening trials. These were preceded by an auditory spoken cue (“Rest”), and no auditory stimuli were presented for the same duration as a normal trial and response period. No “Respond” cue was presented after “Rest” trials. Subjects were instructed to keep their eyes open throughout the listening experiment. A featureless black screen was displayed during the whole experiment and no instructions to fixate were given. Subjects were naïve to the purpose of the experiment, and were told that the eye tracker would be used for a separate visual task that took place between the two blocks of the auditory task.

### Saccade Distractor task

In between the two blocks of the auditory task, subjects performed a visual distractor task that had two components: (1) visual fixation to a central cross (white on black background); and (2) forced saccades to a white cross that appeared unpredictably on each corner of the black screen (see “Eye Tracking” Section). These two tasks lasted 32 s each and were repeated four times. Four rest periods of 32 s duration were interspersed between tasks, wherein a blank screen was presented. The distractor block served as an explanation to subjects for the presence of the eye tracking equipment, for calibration of the eye tracker, and to functionally localize the DAN.

### Eye Tracking

Vertical and horizontal gaze displacements were recorded using a MR-compatible head-mounted infrared camera (Jazz-NOVO, Ober Consulting, Eye movement range—vertical: ±20°, horizontal ±35°; sampling frequency—500 Hz). The voluntary saccades element of the visual distractor task served as a four-point calibration and was performed in between the two runs of the auditory task to be close in time to both runs. In this task, white crosses were presented in each corner of a black 7.5” IFIS-SA LCD screen. The screen was at a viewing distance of 13 cm. The crosses subtended a horizontal angle of 60° and vertical angle of 40° from one another. Gaze displacements to the left, right, upper and lower visual spaces were quantified relative to a center point, which was defined as the average vertical and horizontal gaze position across the run.

Eye movements were analyzed using the Jazz-Manager Software (saccade detection, blink removal) and using in-house software based on MATLAB (normalization and gaze displacement measurement). For each participant, gaze displacement along both axes was detrended to remove low frequency drifts, and normalized by dividing by the standard deviation within each 17 min run. The saccade detection algorithm involved first a differentiation of the raw eye position signal (in degrees of angle) to velocity (°/s). A saccade was then determined if it satisfied all of the following criteria: (i) an initial velocity of 35°/s or greater; (ii) a minimum peak velocity of 100°/s; (iii) a maximum duration of 300 ms; (iv) a minimum duration of 20 ms; (v) an inter-saccadic interval of 50 ms or greater (since very short inter-saccadic intervals of <50 ms would indicate either artifact or pathological eye movements such as ocular flutter or opsoclonus).

The eye blink detection algorithm uses the fact that during an eye blink, the eye moves primarily in the vertical plane, first up and then down (“Bell’s phenomenon”) and hence this algorithm used only the vertical eye signal. It also follows that blinks are bi-phasic with two velocity peaks per blink. The eye blink detection algorithm used the following criteria: (i) a first peak minimum velocity of 200°/s; (ii) a second peak minimum velocity of 100°/s; (iii) a maximum inter-velocity-peak duration of 150 ms; (iv) a total blink duration of between 100 ms (minimum) and 500 ms (maximum); (v) an amplitude of 15° or greater; and (vi) since the vertical eye position at the end of a blink is typically close to the pre-blink position, a ratio of the final to initial vertical eye position for a blink should be close to 1. This ratio was set to between 0.6 (minimum) and 1.4 (maximum).

### MRI Acquisition

MRI data were obtained using a Phillips Intera 3T MRI system with an 8-element phased array head coil and sensitivity encoding. High-resolution (1 mm × 1 mm × 1 mm) T1-weighted whole-brain structural images were obtained for each participant to allow accurate spatial registration of the functional images. Functional MRI data were acquired using an echoplanar imaging (EPI) sequence. Continuous data acquisition was used to collect whole-brain images in 44 axial slices with a slice thickness of 3.5 mm, and a repetition time (TR) of 3 s (echo time (TE) = 45 ms, field of view (FOV) = 220 × 143 × 190 mm, 44 slices, 128 × 128 voxels per slice, voxel size: 2.18 × 2.18 × 3.25 mm). The first three volumes were discarded. A total of 674 whole brain functional images were acquired for each subject, split into two runs of 337 images. Paradigms were programmed using MATLAB and stimuli presented through an IFIS-SA system (*In Vivo* Corporation). Eye tracking data and stimulus presentation were synchronized to the initial scanner pulse. Responses were recorded through a fiber optic response box (Nordicneurolab, Norway), interfaced with the stimulus presentation PC running MATLAB.

### fMRI Image Analysis

Standard preprocessing was carried out using FSL [FMRIB’s Software Library (Smith et al., 2004)]^1^. Image pre-processing involved realignment of EPI images to reduce the effects of motion between scans, spatial smoothing using a 8 mm full-width half-maximum (FWHM) Gaussian kernel, pre-whitening using FMRIB’s Improved Linead Model (FILM) and temporal high-pass filtering using the default cut-off frequency of 100 Hz. FMRIB’s Linear Image Registration Tool (FLIRT) was used to register EPI functional datasets into a standard MNI space using the participant’s individual high-resolution anatomical images. We also ran three separate confirmatory analyses using nonlinear registration and spatial smoothing at 6 and 10 mm to test that our choice of registration and smoothing parameters were not affecting the result. These analyses produced qualitatively similar activation patterns to those reported.

The listening task was divided into four conditions depending on the cue and dichotic/diotic method of stimulus presentation. Seven variables were entered into a general linear model with the onsets and durations of the Cue, Response, Listen-Left, Listen-Right, Listen-High/Low (diotic) and Listen-High/Low (dichotic) trial periods, and one additional Error variable that modeled the trials in which subjects responded incorrectly (across all conditions). The model included the full duration of each condition. “Rest” trials were not modeled in the general linear model and served as the implicit baseline along with the silent periods following each trial. To rule out that the observed behavioral and neuroimaging results might be a result of participants closing their eyes during the trials, we ran a confirmatory analysis where we removed any trials during which no saccades were detected. In this separate confirmatory analysis, no-saccade trials were included in the Error variable and excluded from the task condition variables. This analysis produced similar neuroimaging and eye tracking results. Individual variability in the number of trials without saccades also did not correlate with any of the behavioral rankings (gaze position bias, saccade inhibition or task inhibition). A synthetic double-gamma hemodynamic response function was convolved with each explanatory variable and its first temporal derivative was included to account for variability in the hemodynamic delay function. Six motion parameters were included in the general linear model as confound regressors. To more conclusively rule out motion as a potential confound, we ran a confirmatory analysis using 24 motion regressors which produced qualitatively similar results.

### Group fMRI Analysis

Mixed effects analysis of session and group effects was carried out using FLAME [FMRIB’s Local Analysis of Mixed Effects (Beckmann et al., 2003)]. Final statistical images were thresholded using Gaussian Random Field based cluster inference with an initial cluster-forming threshold of *Z* > 2.3 and a cluster significance threshold of *p* < 0.05. This resulted in statistical maps of clusters significantly activated by the task. Group-mean images were produced by giving each subject equal weighting. Subjects were also ranked by three behavioral variables: (1) mean task performance; (2) mean difference in saccade rate between all task and “Rest” trials; and (3) mean gaze position difference between “Listen-Left” and “Listen-Right” conditions. These rankings were zero-meaned and used as weightings for each subject to probe individual differences in each behavioral measure. These weightings were entered into the same higher-level model. We also carried out a confirmatory group-level analysis using each subject’s (demeaned) *t*-values for gaze position bias and saccade inhibition instead of their relative ranking. This analysis revealed qualitatively similar results to those reported. In the visual distractor task, the fixation and voluntary saccades periods were modeled as separate explanatory variables in a general linear model, with the interspersed rest periods as the implicit baseline. The activations during the voluntary saccade blocks (as compared to the implicit baseline) were used to functionally localize the DAN.

### Functional Connectivity Analysis

To test the network membership of our eye-movement-derived regions of interest, we used resting state data from 20 participants data from the Human Connectome Project (Smith et al., 2013; Van Essen et al., 2013). This dataset was used because of its high quality, and because resting state data was not collected from the participants who took the auditory task. The data consisted of the first 20 subjects from the third phase public release from the Human Connectome Project. Each resting state acquisition was composed of 1200 whole-brain volumes, with a TR = 0.72, collected on a 3T MRI scanner. We used the spatially and temporally preprocessed version of the data. Each run was corrected for spatial distortions from gradient nonlinearity and from motion by registration to a reference image as well as corrected for B0 distortion before being registered to a high-resolution structural image and into MNI standard space. A liberal 2000 s cut-off for a high-pass temporal filter was applied to the data. Twenty-four motion parameters were then aggressively temporally filtered out of the data, along with other non-neural structured noise identified with FIX automatic independent component denoising approach (Salimi-Khorshidi et al., 2014), as described by Smith et al. (2013). In addition, the data was downsampled into 4 × 4 × 4 mm space, to reduce the computational overhead. Functional connectivity (FC) was calculated using the dual regression (Beckmann et al., 2009) pipeline from FSL version 5. Two regions of interest (the activation pattern corresponding to individual variability in either eye gaze displacement or saccade inhibition) were entered separately into the dual regression as the design matrix for a general linear model with the Human Connectome Project (HCP) rest data as the dependent variable. This resulted in a timecourse which was then regressed against the rest data, resulting in a whole-brain spatial map of regression coefficients estimating FC with the initial pattern of activation. The FC map for each subject was then entered into a higher-level general linear model. Results were thresholded using a family-wise error correction for multiple comparisons.

## Results

### Behavioral Results

#### Natural Gaze Position is Biased Towards Attended Sound Location

**Figure 2:**
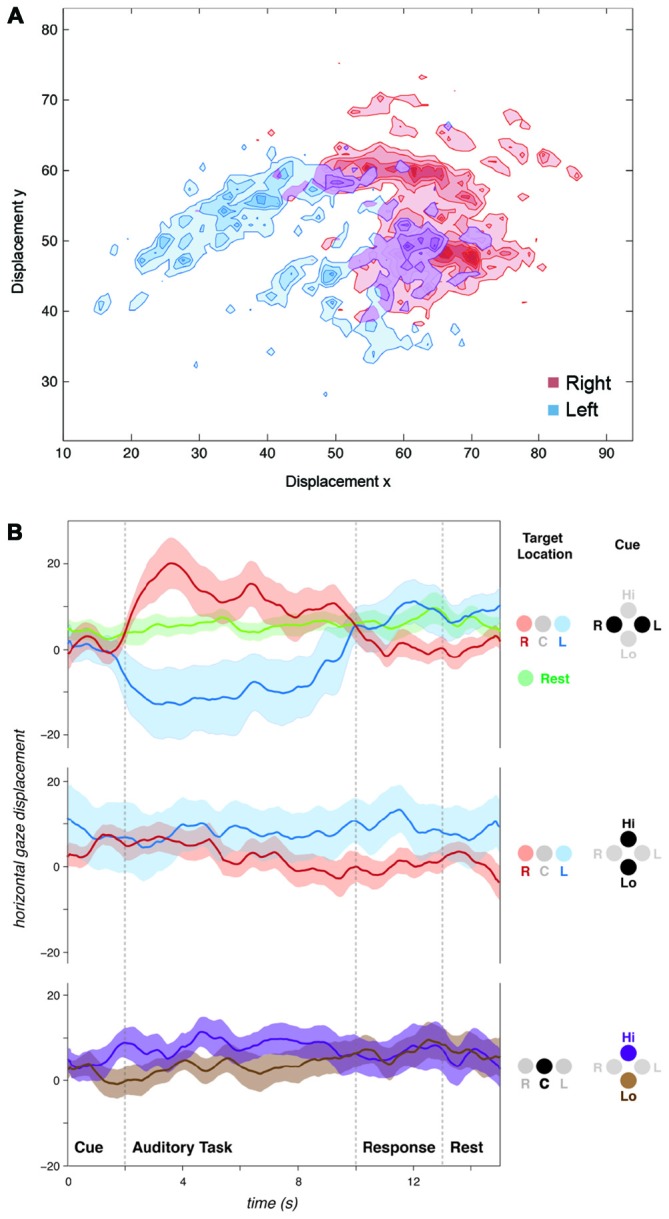
**Auditory spatial attention influences natural gaze position. (A)** Plot of gaze position for a single subject over all spatially cued (“Listen-Left” or “Listen-Right”) trials. Displacement axes: y = vertical, x = horizontal. **(B)** Horizontal gaze position for all subjects grouped by cue and target sound location. Spatial cues influenced gaze position during the subsequent listening task. No gaze biases were observed for spectral discriminations (“Listen-High” or “Listen-Low”) regardless of whether stimuli were presented dichotically (“R” and “L”) or diotically (“C”). Positive values on y-axis represent rightward, and negative values represent leftward gaze displacements. Displacement axes are in arbitrary units.

**Figure 3:**
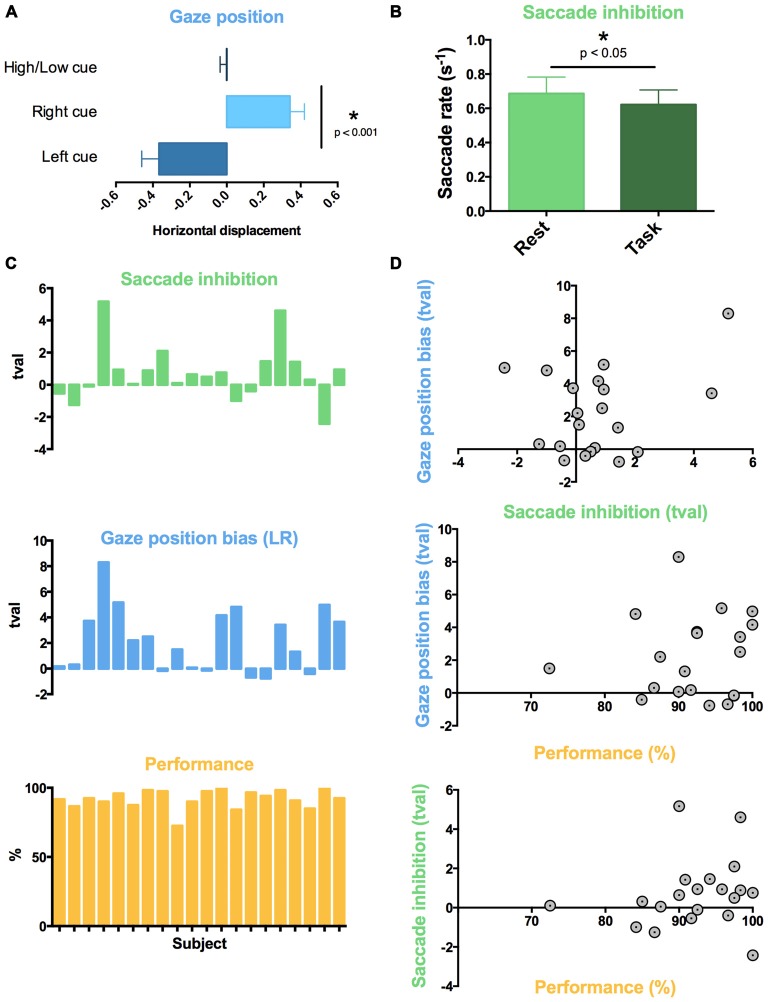
**Individual variability in auditory task-evoked eye movement control and performance.** Across all 20 subjects, **(A)** attentive listening following spatial cues resulted in a gaze position bias towards cued sound, and **(B)** attentive listening reduced saccade rate compared to interleaved rest trials. **(C)** Graphs showing *t*-value (tval) for each subject for the comparison of saccade rate (green; Task vs. Rest) and mean gaze position difference (blue; Listen Left vs. Listen Right Trials), and percent correct responses for each subject (Performance). High inter-subject variability was found in saccade inhibition (all trials) and gaze position bias (spatially cued trials). **(D)** Individual differences in saccade reduction and gaze position bias did not correlate to individual differences in task performance or with each other.

Despite the absence of visual cues and stimuli, following spatial (“Left” or “Right”) auditory cues, subjects tended to shift their gaze left or right consistent with the auditory spatial task (left/right; Figure 2A). A significant difference in mean gaze position was observed between trials when subjects were cued to listen to their left vs. right ear (*t*-test of “Left” vs. “Right” cue trials, *t*_19_ = −4.54, *p* < 0.001, Figure 3A). This gaze position bias was not observed for dichotic trials preceded by a spectral (i.e., “High” or “Low”) cue (Figure 2B). No effects on vertical gaze position were observed for Right-Left or High-Low discriminations (Left-Right: *t*_19_ = −0.42, *p* = 0.68; High-Low: *t*_19_ = 0.25, *p* = 0.81).

#### Saccade Rate Decreases During Auditory Attention

We used a within-subject design to compare saccade rate between task trials and the 10 silent “Rest” trials which were interspersed across each block. The auditory attention task was associated with a lower saccade rate compared to rest (*t*_19_ = 1.973, *p* < 0.05, Figure 3B). This was observed across all trials, regardless of cue type.

#### Individual Variability in Eye Movements and Performance

Subjects performed the task with high accuracy (average 92.1% correct responses, standard deviation 8.3%). No significant differences in performance were observed between spatial (Left-Right) and spectral (High-Low) discrimination conditions (Left-Right: 93.9%, High-Low: diotic 91.4%, dichotic 91.0%, pairwise *t*-tests: all *p* > 0.05, n.s.). Subjects ignored the distractor pitch change successfully in the majority of catch trials (percentage of catch trials with false alarms: 8.2%). In terms of eye movements, high variability in the effect of task was observed across participants for both saccade rate and gaze displacement following spatial cues (Figure 3C). We assessed whether the individual differences in eye movement control predicted individual differences in performance on the auditory task. The task performance scores did not correlate with the inhibition of saccade rate (Figure 3D, *R2* < 0.01, n.s.) or gaze position bias (*R2* = 0.01, n.s.) across subjects. Gaze position bias and saccade rate inhibition across participants were also not correlated with each other (*R2* all < 0.01, n.s.).

### Neuroimaging Results

#### Activity in Frontotemporal and DAN Areas Relates to Auditory Attention and Individual Variation in Task Performance

**Figure 4:**
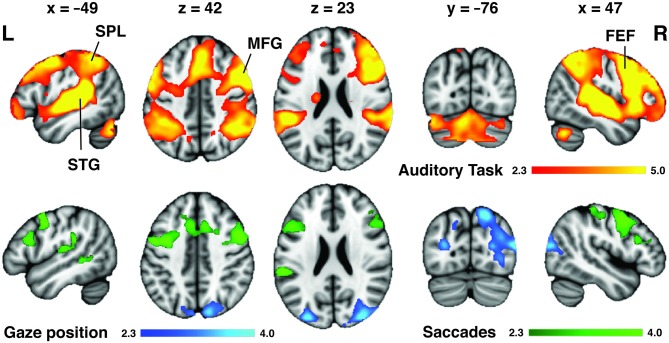
**Neuroimaging correlates of auditory attention task and variability in eye movement.** Orange: across all trials, attentive listening was associated with activation of a widespread auditory network (superior temporal gyri (STG) and sulci (STS) bilaterally) as well as the dorsal attention network [DAN; superior parietal lobes (SPL), frontal eye fields (FEF)] and middle frontal gyri (MFG) bilaterally. However, only the SPL and FEF were associated with individual differences in eye movement control. Blue: subjects that displayed the largest left-right gaze position bias following spatial auditory cues had greater activation of the SPL during the attentive listening periods. Green: subjects that displayed the largest reduction in saccade rate during listening trials compared to rest had greater activation of the FEF and left posterior temporal lobe during the cue period that preceded each trial. Colorbars show cluster-corrected *z*-scores.

Across all task conditions, activation in widespread regions was observed during attentive listening (Figure 4). This network included the SPL, FEF and middle frontal gyrus (MFG) in both hemispheres, as well as both superior temporal gyri (STG) and sulci, and regions of the cerebellum (Table 1). We ranked subjects by their performance scores (%, percentage correct) in the pitch-change detection task (Figure 3C), and assessed which brain regions had activity relating to this rank order during the task. Increased activation of the same widespread DAN and frontotemporal network was associated with improved performance on the task. No differences in the mean (unranked) signal were observed between specific task conditions, such as “Left”, “Right”, “High” or “Low” (diotic and dichotic) trials.

**Table 1:** **MNI coordinates for local maxima of task activation map and individual differences in eye movement maps shown in Figure 4**.

	*x*	*y*	*z*
Auditory task peaks
R STG	70	−28	12
R planum t	46	−32	12
L planum t	−60	−30	8
L STG	−62	−34	10
Saccade effect peaks
R FEF	44	2	54
L FEF	−34	0	44
R-MFG	56	22	28
L MFG	−46	4	52
L STG	−56	−34	26
L p Insula	−50	−22	12
L pMTG	−54	−56	4
Gaze position effect
R Sup Occ	52	−78	16
R SPL	18	−78	50
R Lat Occ	34	−86	24
L Lat Occ	−28	−88	24
L Sup Occ	12	−86	40

*R, right; L, Left; STG, superior temporal gyrus; planum t, planum temporale; FEF, frontal eye fields; MFG, middle frontal gyrus; p, posterior; Sup, superior; Lat, lateral; Occ, Occipital*.

#### Gaze Position Bias Mediated by the SPL

To determine which brain regions mediated the behavioral gaze bias evoked by the spatial task (which were observed during the “Left” and “Right” cue conditions; Figure 3A) whilst controlling for some of the listening requirements and auditory input, we contrasted the spatial and spectral listening conditions (“Listen-Left” + “Listen-Right” > “Listen-High or Low” dichotic). Although no differences were found in the mean signal for this contrast, this may have been a consequence of the individual variability present in gaze position bias (Figure 3C). Therefore, we ranked subjects by their difference in mean gaze displacement between “Listen-Left” and “Listen-Right” trials (average gaze position during all “Listen-Right” trials minus average gaze position during all “Listen-Left” trials). Subjects that showed the greatest mean gaze position difference between left and right trials were given the highest rank, and displayed higher activation of the posterior parietal and superiolateral occipital lobes bilaterally during the spatial compared to spectral listening trials (Figure 4). No differences were found for the direct contrast of “Listen-Left” > “Listen-Right”.

#### Saccade Inhibition Effect Mediated by the FEF

Substantial individual differences in the task-induced inhibition of saccades were also observed (Figure 3B). Therefore, to assess which brain regions mediated this effect, we ranked the participants by the amount of reduction in their saccade rate during the task (saccade rate during listening trials minus saccade rate during rest periods; Figure 3C). Subjects with the largest reduction in saccade rate between rest and task trials were given the highest rank. No regions of activation were observed during the task (compared to the implicit baseline) for this rank analysis. However, during the cue period immediately preceding the task subjects that showed the greatest reduction in saccade rate displayed higher activation of the FEF and MFG bilaterally, as well as some activation in the left STG (Figure 4).

#### Auditory Task-Evoked Eye Movement Regions Overlap with and Dissociate Anterior and Posterior Components of the DAN

**Figure 5:**
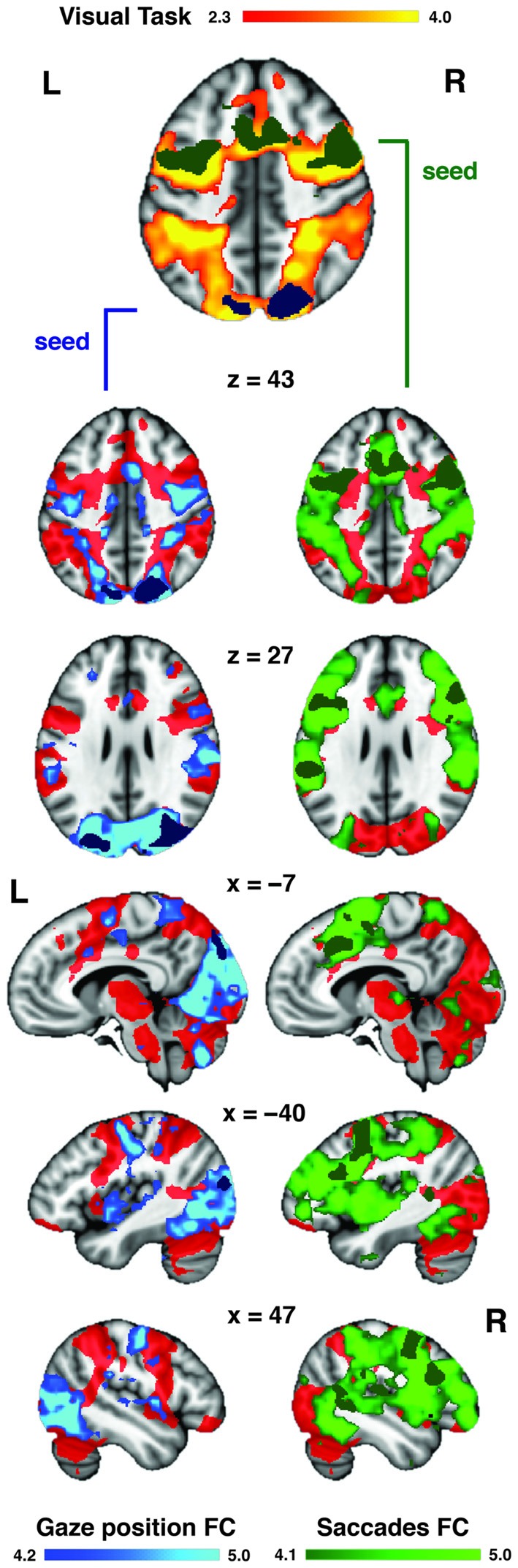
**Auditory task-evoked eye movement regions overlap with DAN.** A visually cued saccade task was used to functionally define the DAN (red) in our dataset. The regions (from Figure 4) associated with increased gaze position bias (blue) and saccade inhibition (green) were used as seeds to assess intrinsic functional connectivity (FC) in the resting state. Both FC maps overlapped considerably with the DAN. The saccade inhibition FC map also revealed stronger connectivity with anterior prefrontal and auditory regions in the temporal lobe. The gaze position bias FC map also revealed strong connectivity with the dorsal and ventral visual streams.

The visual distractor task was used to functionally localize the DAN in our dataset (Figure 5). The activation patterns obtained from the rank analyses of gaze position bias and saccade inhibition were located primarily within regions of the DAN, as evoked by the independent visual task acquired with the same participants. To provide a more detailed description of these two patterns of activation, their FC with the rest of the brain was assessed using resting state data acquired as part of the Human Connectome Project (Van Essen et al., 2013). The regions activated by variability in gaze position were functionally connected to the DAN, including SPL, FEF and SEF near the midline. There was also extensive connectivity with the dorsal and ventral visual streams bilaterally, extending from the SPL via the occipital lobes to the fusiform gyri (Figure 5). Regions activated by variability in saccade inhibition were also functionally connected to the whole DAN, including FEF and SPL, and also the MFG, and visual streams to a lesser extent. There was also extensive connectivity with posterior superior and posterior middle temporal cortices, and anterior regions of the lateral prefrontal cortices. Both FC maps overlapped considerably with the DAN as evoked by the visual task (Figure 5).

## Discussion

The study demonstrates that attentive listening is associated with changes in eye movements that are independent of visual stimuli or visual demands. In addition, this study shows that these crossmodal effects are associated with increased activity in core regions of the dorsal frontoparietal network, the FEF and SPL. Rather than being directly involved in the attentional selection of auditory information, the present results suggest that the role of the DAN during auditory attention is at least partly an indirect one; mediating task-evoked eye movements. It remains to be determined whether the remaining role of the DAN in auditory attention also represents crossmodal process (such as covert multimodal attentional orienting without eye movements, the formation of supramodal spatial maps, or the inhibition of non-auditory inputs) or truly represents direct attentional modulation of auditory information.

Although the neural correlates of eye movements have been investigated previously (e.g., Nobre et al., 2000), to our knowledge, this is the first study that has simultaneously shown that eye movements are evoked by a purely auditory task and that these eye movements are associated with the DAN. This has important implications for the supposed role of the DAN as an amodal attentional system in that it suggests that the DAN mediates crossmodal oculomotor processes, which may or may not be intrinsic to attention to all modalities. Further work is therefore necessary to establish the exact contribution of this brain system to non-visual attention. It is likely that these frontoparietal regions, which are activated under many conditions and have been associated with numerous cognitive processes (Cabeza and Nyberg, 2000), may play multiple roles even within the context of visual and auditory processing.

### Eye Movement Control During Attentive Listening

In this study, participants reduced their rate of saccadic eye movements when they were required to listen attentively (Figure 3B). As no visual cues or stimuli were presented at any stage of the auditory task, and there were no behavioral requirements for eye movements, this natural inhibition of saccades was driven by the requirement to listen attentively. The inhibition of eye movements when attentional resources are required in the auditory modality might serve to reduce the amount of novel incoming visual information which could interfere with the auditory task. Another explanation could be that the resources normally used to plan and execute saccades are diverted to auditory feature selection or spatial orientation during attentive listening. Either way, the auditory-evoked saccade rate inhibition suggests that auditory attention competes with visual control processes during natural listening conditions.

In addition, participants tended to look towards the direction of the cued sound after receiving spatial auditory cues (i.e., to listen to the melodies in their left or right ear; Figure 2). Previous research (Zahn et al., 1978; Zambarbieri et al., 1982) has shown that saccades are often made towards a presented sound, an instance of crossmodal effects during “bottom-up” attention capture. In our study, subjects were presented with sounds in both ears, making it unlikely that “bottom-up” auditory attention capture determined the gaze position bias. This suggests that top-down or endogenous auditory attention, driven by the spoken instructions, influenced gaze position during the present task. Previous behavioral studies have shown that gaze position has a substantial effect on the accuracy of auditory spatial localization accuracy (Razavi et al., 2007; Pavani et al., 2008; Van Grootel and Van Opstal, 2009; Pages and Groh, 2013; Maddox et al., 2014). Subjects with no functioning visual system were found to be severely impaired on a spatial but not a non-spatial auditory attention task (Gori et al., 2014). These findings suggest that auditory spatial maps are likely to be calibrated or fine-tuned by gaze position, meaning that the interaction between oculomotor and auditory processes may be an intrinsic facilitatory mechanism for auditory localization.

### Individual Variability Across Separate Dimensions of Eye Movement Control Reveals Different Listening Strategies

Importantly, although some subjects showed consistent crossmodal effects across trials, not all subjects displayed systematic eye movements during attentive listening. This heterogeneity across participants is consistent with previous behavioral research (Yuval-Greenberg and Deouell, 2011). In the present study, individual variability in saccade rate inhibition was not correlated with individual variability in gaze position bias (Figure 3C), meaning that subjects that displayed one eye movement effect did not necessarily display the other. Therefore, our data suggest that the inhibition of saccades and biasing of gaze position were two separate manifestations of auditory attention affecting eye movements, possibly reflecting underlying listening strategies.

It is possible that the auditory-induced gaze-position bias we observed was facilitatory, helping some subjects to focus on the attended sounds and ignore the competing sounds. However, no relationship between gaze position and task performance was observed in this study. We were therefore unable to distinguish whether gaze position aided performance or was an epiphenomenon. Here, we observed a left-right gaze bias only in trials when subjects received spatial (“Left” or “Right”) cues, not for non-spatial cues (“High” or “Low”), even though these could also be discriminated spatially when presented dichotically. This suggests that the gaze position bias reflected a top-down strategy that some subjects were employing during the spatial task. Although pitch can be discriminated without any spatial information, there is prior evidence for an association between “High” and “Low” pitch and upward and downward visual space (Chiou and Rich, 2012). In this study we did not find any vertical gaze position effects when subjects listened out for different pitch qualities. This could be due to many reasons (outside the remit of this study), such as the nature of the sounds that were used as stimuli, the difference in pitch between high and low competing sounds, as well as the level of musical training of the subjects.

### SPL Activity Correlates with Gaze Position Bias During Spatial Listening

Subjects that displayed the largest difference between leftward and rightward gaze position during spatially cued trials showed the greatest activation in the posterior SPL and superiolateral occipital cortex. This suggests that the posterior SPL plays some role in the spatial orientation of visual fixation during spatial listening. This has implications for studies attempting to isolate the cortical networks supporting auditory attention (Hallett et al., 1999; Shomstein and Yantis, 2006; Salmi et al., 2007, 2009; Kong et al., 2012) as our data suggest that the amount of activation in the SPL corresponds with how much subjects biased their eye movements during spatial listening. It is noteworthy however that the SPL regions associated with spatial gaze bias in the present study did not overlap with the listening task activations (Figure 4), but did fall within the DAN as defined using a visuospatial orienting task (Figure 5). Previous visual studies have shown that gaze position is encoded in the posterior parietal lobe (Williams and Smith, 2010), and crossmodal audiovisual salience maps were located in the SPL (Nardo et al., 2013). It is possible that the SPL mediates the supra-modal formation of spatial maps, partly through the cuing of gaze position (Nardo et al., 2013). The recruitment of the SPL during auditory spatial attention may therefore reflect specifically this cross-modal tuning of spatial maps, indicating that the SPL’s role in auditory attention may also be via the indirect route. The SPL is a candidate for mediating this cross-modal tuning, as it is activated during visual and auditory spatial searching (Corbetta et al., 2008; Hill and Miller, 2010). In addition, both the SPL and FEF are more strongly activated by auditory spatial tasks than auditory tasks involving pitch discrimination (Maeder et al., 2001; Hill and Miller, 2010).

### FEF Activity Correlates with Saccade Rate Inhibition During Attentive Listening

When we probed the brain systems that might mediate crossmodal saccade inhibition effects, subjects that displayed the largest saccade rate difference between rest and task trials showed higher FEF activity during the auditory cue period (Figure 4). These activations overlapped with both the auditory task activations (Figure 4) and the DAN as defined by a visual task (Figure 5). No individual differences in brain activity were observed during the Task period. There are different reasons why this may have been the case. For one, it is not clear whether the inhibition of saccades, or in other words the control of fixation duration should be expected to cause increased activity in higher-order cognitive networks (Henderson and Choi, 2015). The FEF have been proposed to mediate the planning of subsequent saccades (Isoda and Tanji, 2003; Hu and Walker, 2011). As such it is possible that the observed FEF activity in the cue period represents the planning of or preparation for the inhibition of the rate of saccades during the upcoming trial. It is also possible that the analysis techniques deployed were not sensitive to what may be subtle neural correlates of inhibiting saccades. Nonetheless, the finding of elevated FEF activity in those subjects which were prone to showing overt oculomotor differences again suggests that the FEF may be, in part, mediating crossmodal factors during listening, even though this is unlikely to be its only role (Bharadwaj et al., 2014). For example, the FEF shows elevated activity when attention is maintained to spatial locations, even if those locations fall outside the visual field (Tark and Curtis, 2009). Previous work also suggests that the FEF mediates internally guided saccades, while the SPL is involved in both internally and visually guided saccades (Bender et al., 2013).

### The DAN Mediates Auditory Task-Evoked Eye Movement Control

The activation patterns obtained for saccade inhibition and gaze position were located predominantly within the DAN, and each displayed widespread FC with the remaining DAN regions (Figure 5). This suggests that the regions associated with eye control during auditory attention form core parts of the DAN, as shown by their intrinsic connectivity. The gaze position seed in the SPL produced a posterior-loaded DAN which had stronger connectivity with visual regions. The saccade inhibition seed in the FEF produced a more front-loaded DAN with increased prefrontal cortex connectivity, but also interestingly with stronger connectivity to auditory regions in the temporal lobes. One interpretation is that the FEF communicates intrinsically with both auditory and visual regions, which makes it a stronger candidate for an amodal center than the DAN as a whole. However, our results suggest that the FEF influences auditory attention, at least in part, through indirect mechanisms such as the control of eye movements (possibly through suppressing eye movements or altering eye movement planning) rather than through direct top-down control, e.g., the modulation of auditory receptive fields (Fritz et al., 2010). It is possible, although speculative, that the observed auditory effects on eye movement are primarily mediated by the FC between auditory regions and the FEF, which then exerts an effect on the SPL via its strong FC (i.e., the DAN). The SPL may then mediate crossmodal spatial orienting, in part through the cuing of gaze position.

The DAN has been shown to be active under a variety of task conditions, including auditory attention (Corbetta et al., 2008). Further evidence for an auditory role comes from findings that the FEF can show frequency-tagged responses to sounds (Bharadwaj et al., 2014). The present results do not rule out that the DAN plays an important part in attentional orienting to all modalities. Or indeed that the DAN, and in particular the FEF, may have multiple roles. Rather, the present findings suggest that one of the roles that the DAN does perform is to orient the visual system to comply with auditory task demands. This might explain why the DAN is activated during orienting to both auditory and visual stimuli, but is not present during the maintenance of attention to auditory stimuli (Salmi et al., 2007, 2009). This visual orienting process may be intrinsic to auditory orienting, particularly considering how interlinked the auditory and visual systems are (Driver and Spence, 1998), and that there is competition for resources between the systems (Saults and Cowan, 2007). Given that crossmodal processes are likely to play a large part in successful attentional orienting, it is likely that the DAN is crucial for auditory attention. However, in order to establish the DAN’s exact role in listening we propose that the distinction between direct and indirect mechanisms merits further study. For example, this could help make sense of why stroke lesions resulting in visual neglect often do not lead to auditory neglect for spectral features, but do impair auditory localization (Pavani et al., 2002). In this case, damage to the DAN has a dramatic effect on supramodal processes such as spatial orienting, but would not compromise non-spatial auditory processes as its contribution to listening is an indirect, and therefore not necessary one. Thus, the location of lesions within the SPL (and not FEF) would largely determine the selective deficits.

In conclusion, this study shows that auditory attention induces overt eye movements, and that these eye movement effects are mediated by activity in core components of the DAN, the SPL and FEF. Our data suggest that the activation of DAN regions during auditory attention is at least partly attributed to oculomotor control. This is evidence for the DAN being indirectly involved in auditory attention. However, we do not rule out that the DAN plays a larger role in auditory attention, though it remains to be determined whether this remaining role is also indirect (e.g., through covert visual system modulation without eye movements) or direct (e.g., through the modulation of auditory receptive fields). As auditory attention is associated with the inhibition of non-auditory sensory inputs (Langner et al., 2011), it is likely that DAN activation is essential to auditory attention, even if its role is predominantly to modulate and limit interference from visual input. Nonetheless, our data adds to the growing body of evidence (Maeder et al., 2001; Salmi et al., 2007; Braga et al., 2013; Seydell-Greenwald et al., 2013; Michalka et al., 2015) that the role of the DAN in auditory attention is not as clear as in vision. This evidence suggests that there may be parallel but interacting networks for attention to visual and auditory modalities (Salmi et al., 2007; Braga et al., 2013; Michalka et al., 2015), whose mechanisms should be further studied. We propose that the dorsal frontoparietal network mediates intrinsic but crossmodal aspects of auditory attention by virtue of its predominantly visual role.

## Author Contributions

RMB, RZF, RJSW and RL designed the study. RMB, RZF and RL collected the data. RMB, RZF, RL, BMS analyzed the data. RMB, RZF, RL, RJSW and BMS interpreted the results and prepared the manuscript.

## Conflict of Interest Statement

The authors declare that the research was conducted in the absence of any commercial or financial relationships that could be construed as a potential conflict of interest.

## Footnotes

^1^www.fmrib.ox.ac.uk/fsl
